# Parent-Led Stepped Care Trauma Treatment: Parents’ Experiences With Helping Their Child Recover

**DOI:** 10.1007/s40653-023-00537-x

**Published:** 2023-03-31

**Authors:** Else Merete Fagermoen, Tine K. Jensen, Marianne Martinsen, Silje M. Ormhaug

**Affiliations:** 1 Norwegian Centre for Violence and Traumatic Stress Studies, Pb 181 Nydalen, Oslo, 0409 Norway; 2 Department of Psychology, University of Oslo, Oslo, Norway; 3 Faculty of Education, Inland Norway University of Applied Sciences, Hamar, Norway

**Keywords:** Child, Posttraumatic stress disorder, Stepped care trauma-focused cognitive behavioral therapy, Parents, Agency

## Abstract

**Purpose:**

There is a need for interventions for traumatized children that are easily accessible and effective, and that involve parents directly in the recovery process. To meet this challenge, stepped care trauma-focused cognitive behavioral treatment (SC TF-CBT), which consists of a parent-led therapist-assisted intervention as the first step, was developed. Parent-led trauma-treatment is a promising, but novel approach. The aim of this study was therefore to gain knowledge on how parents experience the model.

**Methods:**

Parents who participated in a SC TF-CBT feasibility study were consecutively recruited and interviewed with semi-structured interviews, which were then analysed using interpretative phenomenological analysis.

**Results:**

The parents described that the intervention gave them insights that led to a sense of parental agency. Through the analysis we identified and labelled four themes: (i) understanding my child: how the trauma has affected my child and our relationship; (ii) understanding myself: how my reactions have stood in the way of helping my child; (iii) gaining competence: how to learn specific tasks that were not part of my normal parenting skills; and (iv) receiving support: how guidance, warmth and encouragement was necessary.

**Conclusions:**

The results from this study show how the shifting of therapeutic tasks to parents may facilitate parental empowerment and improve the parent-child relationship. This knowledge may guide clinicians on how to provide support to parents so they can take a leading role in their child’s recovery process after trauma.

**Trial registration:**

ClinicalTrials.gov, NCT04073862. Retrospectively registered 03 June 2019 (first patient recruited May 2019), https://clinicaltrials.gov/ct2/show/NCT04073862.

A large number of children and adolescents experience violence, sexual abuse and other traumas every year (Hafstad et al., 2020; McLaughlin et al., 2013). It is well documented that children exposed to trauma have a heightened risk of developing serious health problems. In particular, heightened levels of posttraumatic stress symptoms (PTSS), depression, anxiety and behavioral problems have been reported in addition to interpersonal and school-related problems (Alisic et al., 2014; Jaffee et al., 2018; Lewis et al., 2019). These serious health problems may result in long-term suffering for the individual and the family (Norman et al., 2012) as well as excessive costs for society (Bellis et al., 2019). Although many children experience rapid decline in PTSS the first months after traumatization, a review of longitudinal studies show that there is little change in symptom recovery after 6 months, indicating the need for interventions (Hiller et al., 2016).

Since many traumatized children develop symptoms that may alter their behaviour, being a parent to a traumatized child can be challenging. Parents might feel a lack of control, helplessness or failure, and normal parenting skills may not be sufficient to meet the new challenges (Elliott & Carnes, 2001). Many parents also experience high levels of distress related to their child’s trauma exposure (Holt et al., 2014; Thoresen et al., 2016), particularly if they are traumatized themselves (Tutus & Goldbeck, 2016). At the same time, studies emphasize the importance of parental functioning and care after traumatic events (Brown et al., 2020; Trickey et al., 2012). There is evidence that parenting that encourages emotional expression, acceptance, positive reframing, and a supportive parent-child relationship may be associated with reduced symptoms for the child, whereas overprotection, hostility and coercive control may have the opposite effect (Scheeringa & Zeanah, 2001; Williamson et al., 2017). Despite the challenges, many parents are motivated to help their children after traumatic events. In fact, one of the most frequently reported barriers to seeking help and completing treatment for their children is the parents’ desire to solve the problems on their own (Thurston & Phares, 2008). In sum, there is a need for interventions that are easily accessible and effective, and that involve the parents and contribute to their ability to help their children.

Stepped care trauma treatment, where parents are responsible for the child’s recovery under the guidance of a therapist, may be one way to meet this challenge. Although parenting skills have been targeted in several parenting intervention programs, with aims of improving maladaptive parenting practices, parent-led treatment programs have a fundamental different approach, where the therapist’s aim is to help the parent treat their own child and not just change their parenting practices. One overall aim in parent-led treatments is thus to increase parental agency. According to Kuczynski and De Mol`s (2015) social relational theory (SRT) of child development, parents and children are seen as agents who actively contribute to development within a relationship context. Agency refers to their active contribution in this developmental process, where they are able to make sense of their environment and make choices within the relational contexts. Development is seen as a result of transactional processes, where both parents and children respond to new emerging features of the other. Past experiences (i.e. experiencing trauma) and future goals (i.e. increasing daily functioning) affect how they interpret, construct meaning, and intervene in the relationship. When a child is struggling, parents may not know how to interpret their child’s behaviour, they may feel helpless and their parenting practices may not be helpful. As the parents struggle, the child may become confused, and the parent – child relationship becomes strained. Helping a parent restore faith in their abilities to help their child may help alter a negative pathway.

Several models for parent-led treatment are being developed and tested with promising results for anxious youth (James et al., 2020). There are, however, few efforts to develop parent-led treatment for traumatized youth. One exception is stepped care trauma-focused cognitive behavioral treatment (SC TF-CBT) (Salloum et al., 2014), that builds on the well-established evidence-based treatment for traumatized children and youth: trauma-focused cognitive behavioral therapy (TF-CBT) (Cohen et al., 2017). The SC TF-CBT model consists of two steps and is designed to be accessible and efficient. The first step is parent-led and therapist-assisted. If the child needs more intensive therapist-led treatment, the child is stepped up to Step Two, which preferably consists of 9–12 sessions of TF-CBT. See method section for further details to explain the steps involved in SC TF-CBT. To date, studies on SC TF-CBT have documented comparable results to TF-CBT in reducing PTSS, and two cost-benefit analyses have estimated cost savings of 53.7% and 51.3% (Salloum et al., 2022; Salloum, Wang, et al. 2016b). Parents have reported overall satisfaction with the treatment. However, 1 in 5 parents were feeling uncertain about leading the treatment themselves, and 15% found Step One to be the least helpful aspect of the treatment (Salloum et al., 2015; Salloum, Swaidan, et al. 2016a). This indicates that parents differ in how acceptable they find the treatment model.

Considering the novelty of parent-led trauma treatment, in particular in light of the heightened level of distress of being a parent to a traumatized child, more in depth knowledge about *what* parents find helpful and/or challenging with the model and *why* is needed. Such knowledge is important to improve efficiency and to guide clinicians and service providers on how to tailor appropriate treatment for different families, and can thus have implications for implementation. This is in line with current trends of task shifting to make evidence based treatment available to more children. In this study, we therefore explored parents’ own experiences of helping their children with their trauma symptoms in SC TF-CBT through in-depth interviews that were analysed using interpretive phenomenological analysis.

## Method

This qualitative study is part of a larger feasibility trial of SC TF-CBT in a low-threshold service that provides short-term treatment for children and their parents in 12 Norwegian municipalities.

### Background

#### The Treatment

SC TF-CBT Step One is a parent-led, therapist-assisted treatment for children where the primary responsibility for conducting the trauma-focused interventions is delegated to the parent under the guidance of the therapist. If the child does not respond well to Step One, they are stepped up to receive TF-CBT. In the original version, both Steps One and Two are provided at the Child and Adolescent Mental Health Services (CAMHS) with the same therapist. We implemented an adapted version where Step One was completed in the municipalities’ first line services as an early intervention and Step Two was completed at the CAMHS. Step One consisted of three in-office therapist-led sessions that was spread out during the first 4–6 weeks, 11 parent-led meetings at home over 6–9 weeks, and weekly phone calls from the therapist to the parent (approximately 15 min each). The three therapist-led sessions provided the child and the parent with information about Step One, including rationale, materials, guidelines, structure, and time commitment, and focused on general support and motivation. In the 11 parent-child meetings, the child and the participating parent worked together at home on tasks from the *Stepping Together* workbook. The tasks in the workbook focused on psychoeducation and coping skills such as relaxation, affective expression and modulation; developing a trauma-narrative; and trauma-focused exposure. Parallel weekly phone meetings offered the parents general support, motivation, and technical assistance if needed. For a complete description of the model, see Salloum et al. (2014). If the child met the responder status, defined as four or fewer DSM-5-defined PTSD symptoms measured by the child-rated Child and Adolescent Trauma Screen (CATS) (Sachser et al., 2022), and was rated as improved, much improved or free of symptoms after completing Step One by the therapist, the child and the parent entered the maintenance phase for six weeks to practice the newly achieved skills. If the child did not meet the responder status, the child and the parent stepped up to Step Two, consisting of a referral to their local CAHMS to receive 9–12 sessions of TF-CBT.

#### Inclusion- and Exclusion Criteria for the Feasibility-Study

Study inclusion criteria for the feasibility-study were as follows: (a) the child was exposed to one or more potentially traumatic event(s) after the age of 3, (b) the child had at least five DSM-5-defined PTSD symptoms (measured by the CATS), including at least one symptom each of re-experiencing and avoidance, and the minimum intensity on each symptom was at least 2 on a scale from 0 to 3), (c) the child was between the ages of 7 and 12 years (which is the age group one of the workbooks has been developed for), (d) the parent and the child were willing and able to participate in the treatment and the parent gave written informed consent, and the child gave oral informed consent without the parent present. Since this is a parent-led treatment, it was important for us to ensure that the child felt safe at home and that the child understood that the parent would be working together with the child to resolve their trauma -related difficulties. Therefore, child consent was obtained in a face-to-face conversation alone with the therapist. Study exclusion criteria were as follows: (a) indications of parent or child psychosis, autism spectrum disorder, mental retardation, substance abuse, severe suicidality, or any condition that would gravely limit the parents’ ability to understand CBT or the child’s ability to follow instructions such as cognitive limitations, (b) the need for interpreter, (c) the parent bringing the child to treatment was the perpetrator, the perpetrator was still living at home, or there was other ongoing traumatization, (d) the child was on a psychotropic medication regime that had not been stable for at least 4 weeks prior to study admission, with the exception of at least 2 weeks for stimulants or benzodiazepines, and (e) the child was receiving concurrent trauma-focused psychotherapy.

### Participants

#### The Interviewed Parents

The parents were consecutively recruited until we reached saturation (Saunders et al., 2018). This resulted in 15 caregivers: 4 fathers and 11 mothers, aged 30–61 years (M = 42, SD = 8.08). All were the biological parents of the participating children; therefore, we use the word parent in the rest of the article. A total of 80% of the parents had experienced potentially traumatizing events (M = 3.9, SD = 3.50), measured by the Stressful Life-Events Screening Questionnaire (Goodman et al., 1998). Their self-reported mean posttraumatic stress level reported pre-treatment was 21.6 (SD = 17.7), as assessed with the PTSD Checklist for DSM-5 (Weathers et al., 1993), with 20% scoring above the cut-off of 33. Their mean symptom level of anxiety and depression pre-treatment, measured by the Symptom Checklist-10 S (Strand et al., 2003), was 1.8 (SD = 0.65), with 47% scoring above the cut off of 1.85. Table 1 provides demographic information about the participating parents.

**Table 1:** Demographics of Parents in Stepped Care TF-CBT (n = 15)

Descriptives	%	*n*
Birth country		
**Norway**	100	15
Living conditions		
Child lives with both parents	47	7
Child lives mostly with mother	33	5
Child lives mostly with father	7	1
Shared custody (equal)	13	2
Education of the participating caregiver		
Mandatory (10 years)	7	1
Upper secondary	14	2
University (minimum 4 years)	80	12
Relationship status		
Single	33	5
Married	33	5
Co-habitating with partner	20	3
Separated	13	2

#### The Children

The children, nine boys and six girls, were on average 9.5 years old (SD = 1.25). The children’s index trauma, as identified by the children on the CATS (Sachser et al., 2022) was as follows: four (26.7%) cases of child maltreatment, four (26.7%) cases of severe and threatening bullying, three (20%) cases of stressful medical procedures, one (6.7%) case of witnessing intimate partner violence, one (6.7%) case of natural disaster, one (6.7%) case of being attacked, and one (6.7%) case of a death of someone close. All children had experienced more than one trauma, and the mean number of traumatic events was 3.7 (SD = 1.44) from a list of 15 potential events. The mean posttraumatic stress level reported by the children pre-treatment was 27.2 (SD = 5.2), as assessed with CATS, where the maximum possible sum-score is 60 and a sum-score of 15 is typically considered a cut-off for clinically significant symptoms (Sachser et al., 2022). One participant dropped out of treatment after two sessions (i.e., after completing two out of 11 home meetings); the rest of the participants completed Step One. Two (13.3%) children were stepped up to Step Two. The remaining 12 (80%) children responded after Step One.

#### The Therapists

The therapists (8) volunteered to participate in the feasibility study and receive training. Seven of the therapists were clinical psychologists, and one was a psychiatric nurse. All of the therapists received four days of initial training, including two days of training with the developer of the treatment, Dr. Alison Salloum. They also completed a certified TF-CBT web-based training program (https://tfcbt2.musc.edu) and participated in monthly consultation calls with Dr. Salloum. All therapist-led sessions were audiotaped, and fidelity was assessed by trained supervisors based on a checklist developed by Dr. Salloum. Model fidelity was high (above 95%), agreement between the ratings was above 95%, and all cases were approved. The therapists were not part of the research team.

#### The Researchers

The research team consisted of a clinical psychologist and doctoral student, a professor in clinical psychology, an associate professor in physical activity and health, and a clinical psychologist with a PhD. All three psychologists have extensive experience with TF-CBT. Together, the researchers have backgrounds in cognitive psychology, developmental psychology, and trauma theory and treatment research.

### Procedure

#### Recruitment

Parents were contacted by e-mail, informed about the study, and asked to participate in interviews 1–3 weeks after they had completed or dropped out of treatment. They were consecutively recruited from the beginning of the trial, and none of the invited parents declined to participate. We pre-decided to determine the final sample by data saturation (Saunders et al., 2018). After interviews of 15 parents, no prominent new themes emerged, and saturation was reached. The decision was made in consensus by the authors after analysing 14 transcripts. The last transcript was read to confirm the decision.

#### Interviews

To explore how the parents experienced SC TF-CBT, we created a semi-structured interview guide consisting of several open-ended questions followed by general prompts to elicit further elaboration (e.g., “What happened”, “Tell me more about that”, and “How did you feel?”) (Table 2). The interview guide was presented to colleagues experienced in TF-CBT and SC TF-CBT for feedback. To ensure that the interview questions were appropriate and acceptable to the parents, we ran two pilot interviews.

**Table 2:** Interview Schedule

Can you tell me what you experienced when assisting your child in parent-led SC TF-CBT?
What did you find helpful/not helpful about the intervention?
Can you tell me about a recent home-meeting?
Is there something about you or your situation that has made it more easy/difficult to complete the intervention?
What is your experience with the therapist?
What do you think about ending the intervention?

The first author conducted all the interviews. She was not involved in providing the treatment but was engaged as a supervisor for four of the therapists treating nine of the cases. Interviews were conducted between April and September 2020. Due to COVID-19, all interviews were conducted through safe and end-to-end encrypted video calls. All interviews were audiotaped with the participants’ informed consent, and all interviews were professionally transcribed verbatim. Interviewing, transcription and analysis took place concurrently.

### Data Analyses

Since we were interested in understanding the parents’ perspectives on their own experiences with SC TF-CBT, interpretative phenomenological analysis (IPA) was used (Smith, 2003, 2011; Smith et al., 2009, 2022). First, IPA is an ideographic approach, with an emphasis on analysing each subject in detail. Second, grounded in phenomenology, IPA is intended to explore people’s experiences, particularly significant events where the daily flow of life is altered. Third, IPA is interpretative; the researcher attempts to understand how the participants make sense of what is happening to them, called a double hermeneutic. In this process the researchers may bring with them both theories and pre-understandings, and in the interest of being transparent, we describe below how the analytical process proceeded.

We analysed the interviews, case-by-case, according to the recommendations made by Smith et al. (2009) and as described in the following: We first familiarized ourselves with the interviews by reading all interviews. We then looked for subordinate themes in the first case. We started by writing down our first impressions, comments and associations and reread the transcript to become as familiar with the content as possible. Then, we transformed the initial notes to phrases to attempt to summarize the essential quality of salient meanings in the interview. Next, we connected the phrases to subordinate themes in the interview. In this process, we looked for connections, similarities and differences between the phrases, trying to cluster them together. We continued this analysis with the other interviews, going through the same procedures for all of them, writing up all the cases separately. We then connected subordinate themes together from the different interviews to higher-level themes. As we made higher level and more abstract descriptions of the themes, we referred back to the interviews to make sure the themes still represented the text.

After familiarizing ourselves with all the data, we discovered how parents constantly returned to talking about how the parent-led model helped them regain a sense of parental agency. They could say things such as “I used to feel helpless”, “I did not know what to do”, and “now I can finally help my child”, reflecting a sense of increased agency throughout the therapy. To inform the further analysis and in an effort to push the field forward from what is already known, we added a particular focus on *how* the model contributed to increased feelings of agency in parents.

### Reflexivity

The first author was familiar with some of the cases through case supervision. This was helpful in the planning and analysis of the study because she knew the context from which the parents were reporting; however, this could also lead to bias in the interpretation of the interviews. Inspired by the Consensual Qualitative Research Model (Hill et al., 2005), we therefore involved all four authors in the analysis to reduce researcher bias and group thinking and enhance validity. The first and second authors formed the primary analytical team. Consensus of the themes was obtained after reading, taking notes, rereading and discussing every interview. The third author also read all of the interviews independently and acted as a discussant. The fourth author read five selected interviews that contained most examples and descriptions to ensure validations of the main themes. Any disparities were discussed until the authors reached a consensus. The results are reported in accordance with standards for reporting qualitative research (SRQR) (O’Brien et al., 2014).

### Ethics

The Regional Committee for Medical and Health Research Ethics and the Norwegian Social Science Data Services approved the main study, and the study was conducted in line with the Helsinki declaration of 1975, as revised in 2008. All participants signed a consent-form. The audio-files and transcripts are stored in a database developed for sensitive data. To ensure anonymity, we have fully de-identified all content and quotes. To personalize the quotes and enhance the readers’ ability to relate to the content, we use fictional names on the participants.

## Results

Many parents described feeling helpless, inefficient and powerless when trying to face their child’s difficulties as they entered therapy. These feelings were often accompanied by feelings of frustration, sadness and shame for not being able to help their child with their emotional difficulties. They wanted to change this and help their child feel safe and happy again, and this was an important motivation for seeking help. Almost all parents described how taking part in SC TF-CBT helped them in the process of helping their child and gaining a sense of parental agency. One of them, Charlotte’s father, said:

I became very enthusiastic when the therapist told me about this therapy because it gave me the opportunity to sit down and talk properly with my daughter…Charlotte has been to therapy before, where therapists have kept me informed about the therapy, but I never really understood how to help until now…. It is important that my daughter realizes how important it is to me that she is okay. Of course, the most important thing is that she gets help, but I don’t send her away to people to talk to because I don’t want to talk to her myself. If I have the opportunity, I’ll do it myself, and this treatment has helped me with just that (father of Charlotte, age 12).

Charlotte’s father wanted to help his daughter but felt he lacked competence. The urgency in his statement might suggest that there is more at stake than being able to give her ordinary fatherly advice. It may be that being able to help his daughter with her trauma symptoms had relational consequences on a more profound level. This parent-led therapy seemed to give him new hope and a new experience of being able to connect with her and help her feel better. Overall, this was a broad experience shared by most of the parents. In the analytical process, we identified that the increased sense of agency was brought about by newly acquired experiences with their child in the home meetings. We identified and labelled the following four themes (i) understanding my child: how the trauma has affected my child and our relationship; (ii) understanding myself: how my reactions have stood in the way of helping my child; (iii) gaining competence: how to learn specific tasks that were not part of my normal parenting skills; and (iv) receiving support: how guidance, warmth and encouragement was necessary. The three first themes also consist of subordinate themes. Table 3 shows the distribution of themes and subordinate themes among the parents. To denote the number of parents expressing a particular experience or idea, the following terms are used: a few (2–3), some (4–6), and many (≥ 7) (Sandelowski, 2001).

**Table 3:** Themes and Subordinate Themes in Parents’ Experiences With Stepped Care TF-CBT From Semi-Structured Interviews

Themes	Subordinate themes
Understanding my child: how the trauma has affected my child and our relationship *n* = 12	The impact of the trauma *n* = 11
	A strengthened relationship *n* = 10
Understanding myself: how my reactions have stood in the way of helping my child *n* = 12	Understanding my own problems *n* = 7
	My reactions on my child’s trauma *n* = 10
Gaining competence: how to learn specific tasks that were not part of my normal parenting skills *n* = 13	Knowing what to do *n* = 13 Knowing when to do it *n* = 9 Knowing why to do it n = 4
Receiving support: how guidance, warmth and support was necessary *n* = 12	

*n* = Number of parents addressing the themes

### Understanding My Child: How the Trauma Has Affected My Child and Our Relationship

Almost all parents highlighted that because they were actively involved in the treatment and were expected to be an important part of the therapy, this helped them to understand their child better. This understanding provided the basis they needed to help their child with their difficulties. They gave two explanations for this: (1) the therapy made me understand the impact of the trauma on my child, and (2) the understanding improved my relationship with my child.

### Impact of the Trauma

All parents were familiar with their children’s trauma experiences before treatment, but in the interviews, it became evident that knowing about the trauma did not necessarily mean they understood how the children were impacted. The home meetings helped many parents understand their children in new ways. Jacob’s (10) mother represents a good example of this. She came to therapy not understanding how Jacob was struggling; their relationship was tense, and he would not open up to her. She said:

If the therapist had done everything, and I had not been present, I would not have gained the insight of how traumatic the experience has been for my son. When he told me about the trauma at our home meetings, I was able to hear and see his expressions first-hand, and that gave me an insight I did not have before. And the more safe he felt, the more he told me, the clearer it became to me, and then I could meet him in new ways (mother of Jacob, age 10).

Being involved in the trauma narrative work enabled her to see, hear and sense the quality and intensity of her son’s emotional expressions as they processed the trauma experience at home as part of the therapy. The involvement gave Jacob’s mother a better understanding of her son’s difficulties that enabled her to act differently to help him, possibly resulting in her being more responsive and allowing him to gradually open up to her. This experience was shared by many of the parents, and several mentioned that they found hope in understanding that the problems did not indicate that they were bad parents or that something was wrong with their child.

#### A Strengthened Relationship

Similarly, some parents pointed out that their newly acquired understanding of their child’s trauma and trauma symptoms strengthened their relationship with their child. This enhanced relationship created new opportunities for parents to help their children. Noah’s mother said:

One of the best things about the treatment is that our relationship is strengthened…and that was necessary for the treatment to work…The way I have noticed this is that we now can talk together with a common set of words. Now he comes to me to explain how he feels, what he thinks, and what he needs. I listen, understand, and respond, and we can have a conversation rather than anger outbursts with him hitting and throwing things around, and me not knowing how to react and giving up (mother of Noah, age 10).

A few parents still lacked an understanding of their child after completing SC TF-CBT, and they found it difficult to help their child. For instance, Lucas’ (7) mother felt that her son had only shared parts of the trauma experiences. This made it difficult for her to differentiate between bad behaviour and behaviour related to the traumas. As a result, she was unsure of how to react and help him in many situations. They were later stepped up for further treatment. This underlines the importance of parents’ trauma-informed understanding of their children’s reactions to be able to help. Many parents described this understanding as a qualitatively new experience offered by the treatment, which gave them hope for change.

### Understanding Myself: How My Reactions Have Stood in the Way of Helping My Child

Many parents also found that talking with their child in the home meetings helped them understand themselves in new ways that were important for their ability to help their children. In particular, they mentioned how their own personal problems, as well as their reactions to their child’s trauma, had stood in the way of helping and that the therapy helped them understand this. This added awareness transformed their own problems and reactions into resources because it enabled them to relate to their child’s difficulties and motivated them to help.

#### Understanding My Own Problems

Half of the parents gave examples of how being so closely involved in the treatment made them more aware of how their own traumas, conflicts, anxiety and depression had made it difficult for them to see their children’s struggles. Mia’s (11) mother explained how she used to avoid talking to Mia because she became upset and how, during the treatment, she became aware that her own issues were related to their shared negative experiences with Mia’s father.

One thing is her reactions, but my reactions to her being angry just like her father are also important. It triggers something in me that makes it difficult for me, it is not necessarily her fault, or the dynamics between us (mother of Mia, age 11).

With the help of the therapist, Mia’s mother was able to recognize her own trauma triggers and differentiate them from reactions to her daughter’s behaviour. The therapist also helped her accept these reactions as normal, and consequently, she became more open, ready and able to help her daughter.

#### My Reactions to My Child’s Trauma

It was not only parents with their own negative experiences who underlined the importance of understanding their own reactions. Many parents also reported how seeing their child hurt evoked strong reactions in them that they needed to understand to be able to help their child. They described how talking about the trauma with their child in the home meetings was challenging, as many children became highly activated, with feelings of anxiety, sadness, irritability and sometimes anger. Jacob’s (10) mother plainly said, “Being there for my son was cognitively and emotionally demanding”.

In line with Jacob’s mother, many parents described how the situation filled them with feelings of guilt, sadness, anxiousness, irritability, and helplessness. For some parents, this had previously led them to avoid the trauma all together to protect themselves and to avoid the feeling of not being able to help, comfort and protect their child. Lucas’ (7) mother said, “I believe you have to be able to dare acknowledge your own feelings related to the trauma in order to have a clarified relation to those feelings…otherwise you end up avoiding your child’s difficulties”. Since she was expected to work so close with her son, Lucas’ mother felt she had to come up with ways to handle her avoidance. Like many of the other parents, she worked on recognizing, understanding and improving her responses. The newly acquired understandings resulted in parents feeling ready and able to be present to help their children with their trauma-related difficulties. Alfred’s (9) mother said, “I am very proud that my son feels more secure. I think that is because he has more confidence that I am able to handle his reactions without becoming so activated myself”. Other parents also underlined how good it felt to stop avoiding, and help and protect their child. Playing such an active part in the therapy process forced them to face their own fears.

### Gaining Competence: How to Learn Specific Tasks That Were Not Part of My Normal Parenting Skills

Nearly all the parents highlighted how SC TF-CBT enabled them to help their child by affording them advice on *how* to help. Their responses can be divided into three main categories: *what* to do, *when* to do it, and *why* do it.

#### Knowing What to Do

In SC TF-CBT, parents and their children work on different tasks, including psychoeducation, relaxation, affective expression and modulation, and trauma-focused exposure. Many parents appreciated learning these tasks. To be able to assist their child in overcoming their trauma-related difficulties, the parents explained how they needed specific and well-described instructions to enhance the competencies they did not possess as a part of their normal parenting skills. For instance, Lucas’s mother found the exposure techniques very useful. She said:

Before the treatment, I never got into a position where I could help him. It didn’t matter what I said or tried. However, when we started the exposure, the anxiety and the stress in his body that used to become too overwhelming, began to diminish. This is what made it possible to get in relation to him again (mother of Lucas, age 7).

Some parents found the tools so helpful that they continued to use them after SC TF-CBT was completed. Theo’s (10) father said, “For instance, yesterday he could not sleep, and then we used progressive muscle relaxation”.

#### Knowing When to Do It

Knowing *when* to use the tools seemed as important as learning the content of the tools. SC TF-CBT has a predefined structure with a regular set of meetings with specific tasks to go through. This structure allows for a well-prepared order of interventions, the time and space to carry them out, and a clear expectation to do so. Many parents highlighted the importance of this structure. Charlotte’s father said:

I liked that we set a frame for the meetings; it made it apparent that now we are in a meeting and then we are not going to do some other things, such as checking the phone...the structure helped me get the conversation started and avoid digressions and distractions (father of Charlotte, age 12).

Another aspect mentioned by a few other parents was the benefit of the structure for gradual exposure to trauma. Having a well-prepared and thought-out plan made both parent and child feel more confident going through the tasks that evoked avoidance.

#### Knowing Why to Do It

In the interviews, some parents pointed out how helpful it was to be provided with a rationale for why these tasks were necessary. Having a sense of *why* helped them stick to the protocol even when the child became emotional. Sebastian’s (8) mother explained how the rationale was necessary for her being able to complete the exposure with her son who had experienced bullying: “Drawing the school made him scream, his entire body became tense, and I became afraid. Then I reminded myself: ‘Now we are repairing’. We continued, he calmed down, and I was able to help him”. The majority of the parents experienced helping their child with trauma-related difficulties to be outside their normal parenting skills. Consequently, it seemed important for them to specifically learn how and why to do the tasks in the intervention. For many, the enhanced competence resulted in their child trusting them to help and finding it worthwhile to come to them for help and support, even after the therapy had ended.

### Receiving Support: How Guidance, Warmth and Encouragement Was Necessary

In the interviews, many parents underlined the importance of the therapist’s support for being able to help their child and complete the therapy. Nearly all parents reported how their relationship with a therapist who conveyed belief in their abilities to do the trauma-related work at home had been crucial for following the protocol. Noah’s mother said:

Part of me was afraid of starting this parent-led therapy because of my fear of being judged or not being able to complete….so it felt very safe to have a therapist I could speak openly with, who gave me lots of confirmations and supportive comments, so that I knew I did things right. Because I felt very insecure (mother of Noah, age 10).

In fear of being judged as a bad mother by a professional, this mother nearly dropped out before she even started. Her confidence in helping her son with his trauma difficulties turned out to be dependent on the therapist’s reassurance and support.

Some parents gave examples of the significance of the therapist’s warmth in listening to them, showing that they cared, normalizing their feelings and offering faith in them and hope in change. Sebastian’s mother said:

When I felt I totally failed, that anybody, even a random woman in the store, would have been a better mum than I, she was so good at supporting me, telling me that this was a normal feeling, that the trauma work we did was hard. She really conveyed belief and hope in me, and that was so uplifting…And she cared. This has been so important, critical I would say (mother of Sebastian, age 8).

Not all parents experienced the therapist being as supportive as they needed them to be. A few parents mentioned that better guidance would have improved their ability to help their child with their trauma difficulties. For instance, the mother of Emma, age 8, who had shared experiences of violence with her daughter, perceived the therapist as inexperienced and stressed, leading to a lack of confidence in the therapist’s ability to help and support them.

I wanted treatment for my girl and help for how I could handle it myself, but I wanted someone older, who had worked longer, more balanced. The therapist felt stressed out, it seemed like the treatment was new to her (mother of Emma, age 8).

The therapist was not able to provide the security this mother needed to help her daughter, and she said this was one of the main reasons for her dropping out of the treatment. This underlines the importance of the therapist’s support, particularly for vulnerable parents.

Lastly, many of the parents mentioned the importance of a good relationship between the child and the therapist. Lucas’ mother said:

Even though he has been angry with her, I know that he liked her from the first time they met, and that has been critical for completing the therapy….she made him feel that he mattered, she listened to him and tried to help him (mother of Lucas, age 7).

## Discussion

Being a parent to a traumatized child can be challenging (Holt et al., 2014; Thoresen et al., 2016; Tutus & Goldbeck, 2016); however, data from the present study suggest that taking part in parent-led, therapist-assisted treatment such as SC TF-CBT can enable parents to overcome these challenges and feel empowered to help their child with trauma-related difficulties by enabling agency resources. These findings are important because several studies have documented the importance of parents’ functioning and support after traumatic incidents for a child’s recovery process (Brown et al., 2020; Scheeringa & Zeanah, 2001; Trickey et al., 2012; Williamson et al., 2017).

According to SRT (Kuczynski & De Mol, 2015), development is a process of constant adaption and change to new emerging features in children and their surroundings. Life circumstances, such as trauma, are an important factor that can bring about behavioral and emotional changes in children. In these situations, both children and parents try to interpret and construct meanings from each other’s behaviour. Sometimes, this results in misunderstandings, conflicting perspectives, needs and goals. This can be, for instance, a conflict between a therapeutic need for exposure and a caregiver’s wish to protect their child from the negative feelings exposure may create, or parents expecting age-appropriate independence and not understanding the need for comfort and support after a traumatic event. Within the constraints of their relationship, they try to solve these tensions by making sense of the contradictions. As a result, accommodations and changes in thinking and behaviour can occur. For this dialectical process to be constructive for development, both children and parents must feel connected and that they matter to each other. Most of the parents we interviewed described the opposite, feeling helpless and powerless towards their child as they entered therapy. Furthermore, they described difficulties communicating with their child and high levels of conflicts at home. According to SRT (ibid.), this happens when agents become isolated rather than connected: they feel misunderstood, rejected and hurt by each other. Often, this may result in coercive, rather than responsive and supportive parenting. As several studies indicate, recovery is associated with supportive, rather than hostile and coercive parenting (Scheeringa & Zeanah, 2001; Williamson et al., 2017); therefore, in order for parents and children to connect, it would seem particularly important to help parents understand their children in light of traumatic events and enable them to actively help their child. Interestingly, apart from being able to help their child, the primary motivation for parents to join SC TF-CBT was to better connect with their children again. In accordance with SRT, it seems important to focus on how to facilitate the dialectic transactions and improve the connection between child and caregiver. This again may facilitate the parents’ agency in helping their child, through what Kuczynski and De Mol`s (2015) call three resource domains: Individual, relational and cultural resources.

### Individual Resources

To increase their agency, parents have individual resources, consisting of their expertise, information, the ability to think ahead, and to set goals to rely on (Kuczynski & De Mol, 2015). According to the interviews, the treatment seemed to strengthen parents’ individual resources by providing first-hand experiences of the impact of the trauma on their children and an understanding of how their own problems had stood in the way of helping their children as well as by giving them new expertise on how to help.

The analysis suggests that the treatment helps parents gain information that they need to be able to help their children. For instance, a better understanding of the impact of the trauma on their child gave parents better opportunities to interpret and construct meaning of their child’s behaviour. Behaviour, which was previously interpreted as bad behaviour, was, for example, now to a greater degree interpreted as reactions to trauma triggers. This information gave parents new opportunities to accommodate, accept, and support their child, which is considered important to help traumatized children (Scheeringa & Zeanah, 2001; Williamson et al., 2017). According to Kuczynski and De Mol (2015), another important aspect of individual resources is executive power. Gaining new knowledge about how the child was affected by the trauma led to a better ability to think ahead and plan, knowing which situations could lead to trauma triggers and how their child would probably react. This enabled parents to act proactively, being present and supportive in these situations, to prevent future difficulties such as conflicts. According to SRT, the result of this is a better connection between the child and parent and an improved relationship, which is what the parents reported.

The treatment also contributed to an increased understanding of how the parents’ own personal problems and reactions to their child’s trauma had stood in the way of helping their child. This seemed particularly important for parents who shared a trauma with their child. Being expected to be an active part in their child’s treatment, parents were forced to face their own challenges, which made them recognize how they previously had been too absorbed by their own painful experiences to see their child’s difficulties. Active participation also enabled them to differentiate between their own challenges and their child’s challenges. Many parents experienced how they had previously been avoiding their child’s difficulties and how this disconnected them from their child and increased their child’s difficulties. This is in line with studies that emphasize the association between encouraging emotional expression in traumatized children, rather than avoidance, for symptom reduction (Scheeringa & Zeanah, 2001; Williamson et al., 2017). The avoidance also resulted in parents missing out on their children’s contributions in trying to explain their struggles and their attempts to solve them. This newly acquired personal understanding made many parents take better care of themselves, and for the majority, this resulted in being more attentive, present and ready to help their child. In fact, many of them experienced how their own vulnerability could be transformed into a resource, enabling them to better relate to their children and motivating them to help. This points to the need for therapists to make room for parents’ understandings of their own reactions to be able to help their child. Further studies on how parents’ own problems may predict outcome in the treatment, are needed.

### Relational Resources

The analysis showed that during the therapy, the parents made use of relational resources, referring to access to support and help from the therapist. The analysis suggests that the parents went to great lengths to help their children, many working through the intervention in spite of struggles with their own feelings, the child’s avoidance, time pressure, and prioritizing the interventions over other commitments. When the challenges became more than the parents could handle on their own, they sought support. The support made it possible for the parents who felt they failed, or were afraid of failing, to keep going. It gave them the motivation and hope they needed to be able to help their children despite the challenges they experienced. When parents encountered situations, where a child displayed strong emotions they were unable to regulate, support and advice from the therapist seemed essential to proceed. Many parents also gave examples of how therapist support was important for accepting and regulating their own feelings that came up when they worked on trauma-related issues with their children and that this was crucial to stop avoiding the trauma-related difficulties of their children. The descriptions offered by the parents who lacked therapist support and, as a result, struggled to help their child further underline the importance of relational resources. They describe how both a lack of guidance and a lack of support when facing difficult emotions such as shame, guilt, fear, sadness, anger and hopelessness gave them a feeling of being overwhelmed by the intervention. Without being able to trust the therapist to help them if the tasks were beyond the scope of their individual actions, it was difficult to proceed with the intervention.

A general impression from the analysis, which needs further exploration, is that the need for support from the therapist is no less important in parent-led than therapist-led treatment. Rather, it seems that the therapist has to be present and supportive for both the parent and the child, knowing they have less time to get to know them, and therefore must focus on how they actively can use their skills to build a good therapeutic relationship in a shorter amount of time.

### Cultural Resources

Parents’ cultural resources include rights and constraints by practices, customs, concepts and laws of a society (Kuczynski & De Mol, 2015). Many parents come to therapy affected by cultural representations that may not be helpful. One that was shared by many of the parents was that they were to be blamed when their child was struggling, both for not protecting their child from the trauma and for not being able to help their child. Many worry if they would make things worse, even harming or re-traumatizing their child by bringing up painful memories or shameful feelings in their child, or planting false memories in their child by talking about what happened. Furthermore, parents were influenced by the cultural representations that the children’s trauma-related problems had to be solved by an expert alone. All of these representations were described as inhibiting parents’ agency. The essence of SC TF-CBT violates these cultural norms by meta-communicating that parents are capable of providing help themselves. Being included by having an important role in the intervention, trusted to be able to use the capacities they already possessed and to learn new understandings and skills, seemed to shift their representations from blaming to competence, encouraging their agency. It would seem important for therapists to address unhelpful cultural representations that may be inhibiting parents’ agency. This may have implications for implementation, and is important to further study.

### Strengths and Limitations

To our knowledge, this is the first study to explore how parents experience SC TF-CBT, by an independent research group. All interviews were conducted by the first author. This was helpful in the analysis since she had first-hand experiences with the parents and how they spoke of the therapy, but this could also increase researcher bias; therefore, great effort was put into ensuring a reflective research process, including the use of a reflective researcher diary, frequent team meetings and by involving all authors in the data analysis (Hill et al., 2005). The group of parents is less diverse in terms of education and ethnic minority representation compared to the general population in Norway, with 80% of the participants versus 58% of the general population having a university degree, and no parents versus 15% being of an ethnic minority. However, 80% had experienced their own traumas, which may be representative of this group. This study cannot speak to the children’s experiences, and future studies should include child interviews to learn about the children’s perspectives and how this treatment may support them in their recovery. Furthermore, follow up studies on these parents and children will be key to understanding the long-term benefits of this novel intervention. Finally, this qualitative study cannot answer questions of effectiveness of SC TF-CBT. To establish this knowledge, future studies will need to replicate the findings of Salloum et al. (2016b, 2022).

## Conclusion

By taking part in SC TF-CBT, the parents seem to have acquired an increased understanding of their children and themselves. They explain how they gained competence and made use of their personal and professional social networks. Taken together, this seemed to have helped them gain agency and feel empowered to help their children with trauma-related difficulties and to reconnect with their children. Interestingly, even parents who had experienced trauma themselves reported feeling empowered and able to help their child. This is in contrast to common beliefs that parents’ own trauma symptoms may hinder their ability to provide parent-led treatment. Although the therapist has a less prominent role in the therapy than in other therapy modalities, the importance of a strong alliance with both parents and children should not be underestimated. Moreover, including both parents in the model may be helpful. Finally, parents’ reflections indicate that providing therapy that helps them access their agency resources; personal, relational and cultural, and restore the relationships with their children may have long-lasting effects on the children’s development, but this needs to be examined empirically.

## Data Availability

Processed data for this study can be shared upon request, by emailing the corresponding author.
